# Efficacy and safety of an antiviral Iota-Carrageenan nasal spray: a randomized, double-blind, placebo-controlled exploratory study in volunteers with early symptoms of the common cold

**DOI:** 10.1186/1465-9921-11-108

**Published:** 2010-08-10

**Authors:** Ron Eccles, Christiane Meier, Martez Jawad, Regina Weinmüllner, Andreas Grassauer, Eva Prieschl-Grassauer

**Affiliations:** 1 Common Cold Centre, Cardiff School of Biosciences, Cardiff University, UK; 2 Marinomed Biotechnologie GmbH, Veterinaerplatz 1, A-1210 Vienna, Austria

## Abstract

**Background:**

The common cold, the most prevalent contagious viral disease in humans still lacks a safe and effective antiviral treatment. Iota-Carrageenan is broadly active against respiratory viruses in-vitro and has an excellent safety profile. This study investigated the efficacy and safety of an Iota-Carrageenan nasal spray in patients with common cold symptoms.

**Methods:**

In a randomized, double-blind, placebo-controlled exploratory trial, 35 human subjects suffering from early symptoms of common cold received Iota-Carrageenan (0.12%) in a saline solution three times daily for 4 days, compared to placebo.

**Results:**

Administration of Iota-Carrageenan nasal spray reduced the symptoms of common cold (p = 0.046) and the viral load in nasal lavages (p = 0.009) in patients with early symptoms of common cold. Pro-inflammatory mediators FGF-2, Fractalkine, GRO, G-CSF, IL-8, IL-1α, IP-10, IL-10, and IFN-α2 were reduced in the Iota-Carrageenan group.

**Conclusions:**

Iota-Carrageenan nasal spray appears to be a promising treatment for safe and effective treatment of early symptoms of common cold. Larger trials are indicated to confirm the results.

## Background

Common cold is the most prevalent contagious viral disease in humans. It is caused by a variety of viral pathogens with human rhinoviruses (HRV) being the most abundant ones. Affecting the upper respiratory system, symptoms like blocked nose, cough and sneezing are most common [1,2]. The socioeconomic losses associated with viral respiratory tract infections, however, are huge [3,4] with enormous direct and indirect costs for our health care system [5]. Colds also pose a threat for the very young or old, ailing and/or high risk groups like immunocompromised patients, COPD patients, asthmatics or lung transplant recipients [1,6]. A wide range of remedies is sold on prescription and over the counter, but evidence-based medicine systematic reviews conclude that there is still no reliable prevention or cure available and potential serious side effects of popular products also have to be considered. Given the multiple causes of common cold, the Cochrane collaboration suggested to focus future research efforts on non virus-specific compounds [7]. Effective formulations containing antiviral agents are needed for the safe and efficacious treatment of common cold symptoms and the containment of viral propagation. Potential side effects should also be minimal due to the usually nonhazardous nature of the indication.

Carrageenan is a sulphated galactose polymer, derived from Rhodophyceae seaweeds. It is commonly used in food preparations and topical products for its gelling and emulsifying properties. The three main copolymers are designated as Iota (ι), Kappa (κ) and Lambda (λ), depending on the number and location of sulphate moieties on the hexose scaffold structure [8]. Carrageenan compounds are on the US Food and Drug Administration "Generally Recognized as Safe" (GRAS) list of products for consumption and topical applications [9]. In the pharmaceutical industry, carrageenans are used in topical formulations at daily dose levels up to 2% (~30 mg/person).

In a recent publication, we presented our in-vitro findings on the antiviral properties of Iota-Carrageenan against HRVs (11), which add proof to previous findings on antiviral properties against other viruses [10-13]. The presented exploratory study was designed to determine the magnitude of any effect of Iota-Carrageenan nasal spray on the severity of common cold symptoms relative to placebo treatment. The secondary objective was to analyse effects on biomarker levels and presence of common cold viruses in nasal lavage samples.

## Methods

### Subjects

Healthy volunteers > 18 years, who were briefed in advance and signed the consent declaration prior to any study-related procedures, were recruited for the study. Anonymity was guaranteed as study data and information on subjects was kept safe to prevent communication to third parties. Subjects were free to withdraw from the study at any time without prejudice to further treatment.

The following symptoms were assessed with inclusion into the study and on each study day: local symptoms were sore throat, blocked nose, runny nose, cough, sneezing and systemic symptoms were defined as headache, muscle ache, and chilliness. Symptoms were assessed on a 4 point scale: 0 = none (symptom not present in previous 24 h), 1 = mild (sensible, but not disturbing or irritating), 2 = moderate (symptoms sometimes disturbing/irritating), 3 = severe (symptoms disturbing/irritating most of the time). This scoring system was developed by Jackson in 1958 and is widely used in common cold clinical trials[14]. In order to recruit subjects with early onset of common cold, on study entry, subjects had symptom scores of 1 or greater for sore throat, runny or blocked nose and a total symptom score of 9 or less for the sum of severity scores comprising headache, muscle ache, chilliness, sore throat, runny nose, blocked nose, cough, and sneezing. Study participants agreed to refrain from taking any other medications intended to prevent, intervene with or treat coughs/colds/flu-like symptoms, starting with study entry and continuing through day 7.

Entrants were excluded from the study for the following predefined reasons: unwilling to sign the consent form, a known hypersensitivity or allergy to any component of the study medication, a clinically significant cardiovascular, endocrine, neurological, respiratory, or gastrointestinal disease or history, or any other current disease that was considered by the investigator as an exclusion criteria, e.g. current allergic rhinitis, chronic obstructive pulmonary disease (COPD). Although not explicitly mentioned in the study protocol asthma was such an exclusion criterion and no patients with asthma were recruited. Furthermore, subjects were also excluded by the investigator if a severe nasal septum deviation or other condition was present that could have caused nasal obstruction, such as nasal polyps or nasal/sinus surgery in the past, and influenced symptom scores. Additionally, participants with a history of alcohol/substance abuse or on prescription medication/concomitant therapy other than for contraception, e.g. systemic steroids, intranasal medicines, antibiotics, were also excluded by the investigator. Further reasons for exclusion were incidence of common cold or flu like symptoms for more than 48 h, current smoking, relationship to any study personnel, and administration of any investigational drug or participation in any other clinical trial within 4 weeks of entry into our study. All co-existent diseases or conditions were to be treated in accordance with prevailing medical practice.

### Study design and objective

**Figure 1:**
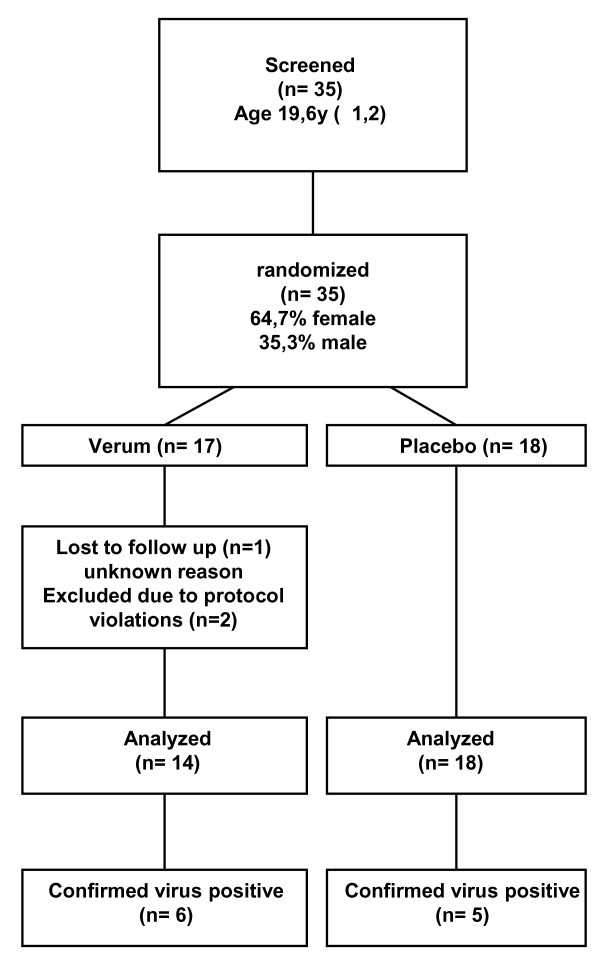
**Flow diagram for study participants including demographic data**.

The current study was designed as a single centre, randomised, double-blind, parallel group, placebo-controlled comparative survey in subjects with early symptoms of common cold to assess the efficacy of a 0.12% Iota-Carrageenan nasal spray in the early treatment of natural colds. Chosen research design, control groups and variables assessed are standard for this field of research, as are the ordinal scales used. A study flow chart is shown in Figure 1.

Subject randomization was done following verification of inclusion/exclusion criteria. Neither investigators nor subjects knew the assigned treatment. Randomization was performed by providing a each test nasal spray with a unique code number. Randomized subjects were assigned a nasal spray at visit 1 (treatment day 1). Subjects completed a daily diary of common cold symptom scores over 7 days and underwent nasal lavage on day 1 before the first treatment and on days 3 or 4. Nasal dosing of study medication was 3 ×/day for 4 days.

The study was conducted between February and May 2008 at the Common Cold and Nasal Research Centre, Cardiff School of Biosciences, Cardiff University, UK. Symptoms and application of treatment was documented in the patient diary. Coding was performed by CRO Bioconsult GmbH (Perchtoldsdorf, Austria) and decoding after trial review and data base lock.

This study was performed in compliance with the ICH E6 Note for Guidance on Good Clinical Practices (CPMP/ICH/135/95)5, and the principles of the Declaration of Helsinki, local drug law and standard operating procedures of the investigator, sponsor and CRO involved. The study protocol and attached documentation (consent form, subject information, CRF etc.) were approved by the responsible South East Wales Local Research Ethics Committee[15]. The signed study protocol was available to the journal editor during the reviewing process. This trial was registered in the European clinical trial registry under the Eudract Number 2007-007577-23.

### Study endpoints

The primary efficacy measure was defined prospectively as the mean total symptom score (TSS) for study days 2- 4 with TSS being the sum of 8 individual symptom scores as described above. Maximum TSS score was 24 for each recorded time point. For statistical analysis, TSS of study days 2- 4 were summarized per subject and individual means calculated.

The secondary efficacy measure was defined prospectively as TSS on separate study days 1/2/3/4/5, as the mean total systemic symptom score (TSSS) (headache, muscle ache, chilliness) for study days 2- 4, and TSSS on separate study days 1/2/3/4/5. Further secondary efficacy variables were local symptom score (LSS) (sore throat, blocked nose, runny nose, cough, sneezing) mean of study days 2- 4, and on separate study days 1/2/3/4/5, and individual symptom scores (ISS) (headache, muscle ache, chilliness, sore throat, blocked nose, runny nose, cough, sneezing) on separate study days 1/2/3/4/5.

Exploratory efficacy variables included virus detection/total viral load, cytokine expression detected in nasal lavage and subject acceptability of test nasal sprays using a visual analogue scale (VAS, 1- 10). Furthermore, data on subjects' willingness to use the product in the future via an ordinal scale (strongly agree, agree, disagree, strongly disagree) were collected.

### Study medication and dosing

Iota-Carrageenan nasal spray (Verum; unlabelled Coldamaris prophylactic^®^) 1,2 g/L, NaCl 5 g/L, water for injection [WFI] ad 20 ml 20.4 g) and placebo (NaCl 9 g/L, WFI ad 20 ml 20.4 g) were manufactured by MoNo chem-pharm Produkte GmbH (Vienna, Austria). Spray solutions were clear, colourless, odourless and free of particles. Verum and placebo nasal sprays were identical in shape, size and colour to allow a double-blind design, and were randomized at the CRO. Before administration, the spray was to be shaken and primed until a fine mist was delivered. One spray application of 140 μl was delivered to each nostril 3 ×/day for 4 days. On day 1, the first application was taken at study entry (9 a.m. to 5 p.m.), the next two applications of the spray were equally spaced through remaining waking hours on day 1. Nasal spray applications on days 2/3/4 were administered equally spaced during waking hours. To control compliance with the study protocol, returned spray bottles were weighted and weights compared to weights on dispensing, thereby evaluating the weight of medication taken over the study period. Subject No. 10 (placebo) missed the first dose on day 1 and 4, the third dose on day 2, and therefore took only 75% of planned doses. All other subjects had 100% compliance according to the diary documentation. In total, 99.3% compliance was reached in this study. Dependent on the time point of inclusion in the study, the expected range of difference in weights was between 2.80 g and 3.36 g. Mean difference in the verum group was 3.12 g (± 0.84 g), and in placebo group 3.84 g (± 1.85 g) concluding that more placebo than verum doses were administered. This result supports the good compliance reported by the subjects in the diary.

Onset of common cold symptoms (days before visit 1) is displayed in Additional file 1: Table S1.

### Analysis of nasal lavages

Nasal lavage was performed on day 1 (study inclusion) and on days 3 or 4. Samples were collected by instillation of 5 ml of 0.9% sterile saline into each nostril, washes expelled into waxed paper cups, samples pooled (per subject) before processing at 4°C within 3 h. 8 parts lavage were mixed with 1 part 5% bovine albumin (Sigma Aldrich, Vienna, Austria) and 1 part 10× protease inhibitor cocktail (Roche, Germany), portioned to 5 samples, and stored at -80°C until further analysis.

For the determination of respiratory virus load and virus identification in nasal lavage, real time PCR analysis was performed for influenza virus type A + B, respiratory syncytial virus type A + B, parainfluenza virus types 1/2/3/4, coronavirus types OC43 and 229E, rhinoviruses (major/minor group viruses), human metapneumovirus. Assays were performed using QiAamp Viral RNA Mini and Qiagen QuantiFast Probe PCR Kits (QIAGEN GmbH, Hilden, Germany), RealAccurate Respiratory RT PCR Kit (Pathofinder, Maastricht, the Netherlands) and Real Time PCR 7900 HT Sequence Detection (Applied Biosystems, Foster City, USA) according to the manufacturer's instructions. This setup enables detection of 12 RNA viruses that account for approximately 90% of respiratory tract infections. In brief, 140 μl samples were thawed on ice, transferred to 560 μl Buffer AVL with carrier RNA, spiked with 10 μl internal control, and eluated RNA was stored at -20°C for a maximum of 4 hours until transfer to -80°C. RT-PCR reaction was performed in a final volume of 20 μl. The following real-time cycler conditions were used: 30 min reverse transcription at 50°C, 15 min activation of Taq DNA polymerase and inactivation of reverse transcriptases at 95°C, 15 s denaturation at 94°C, 60 s annealing and extension at 55°C, 42 cycles. The ct values of all positive samples were anti-logged on the basis of 2. The resulting values of virus positive samples from the first visit were set 100%. The relative quantity of virus positive samples at the second visit was calculated in percent of the value of the first visit.

Lavage samples were assayed in duplicates for measurement of relative cytokine quantity. Cytokine concentration was determined by Milliplex MAP Human Cytokine/Chemokine Kit 96 Well Plate Assay (Millipore Corp., St. Charles, USA) according to the manufacturer's instruction. 0.9% NaCl was used as matrix complying with lavage medium and the following cytokines measured: IL-1 α, IL-1 β, IL-2, IL-3, IL-4, IL-5, IL-6, IL-7, IL-8, IL-9, IL-10, IL-12 p40, IL-12 p70, IL-13, IL-15, IL-17, EGF, Eotaxin, Fractalkine, G-CSF, GM-CSF, IFN-γ, IP-10, MCP-1, MIP-1 α, MIP-1 β, TNF-α, FGF-2, GRO (includes GRO alpha, beta and gamma), IFN-α2, IL-1ra, MCP-3, MDC, sCD40L, VEGF. Fluorescence was determined in a Luminex 100 reader and data analysed by Luminex software (Riverside CA, USA) using a 4- parameter logistic curve fitting method. The lower quantification limit was 3.2 pg/ml, values below were set to 'zero'. Duplicates that resulted in only 1 detectable concentration were omitted from analysis.

### Statistical analysis

Comparisons between the groups were done by means of Mann-Whitney U-tests for continuous variables and by means of Chi square tests for ordinal or nominal distributed variables. Tests between treatment groups were performed by Mann-Whitney U-tests. For tests within groups, the Wilcoxon matched pairs signed rank test was used. The trial was designed as an exploratory study. The magnitude of any effect of the nasal spray on common cold symptoms was unknown. Based on the experience of the study centre it was calculated that a study number of 30 healthy subjects should provide sufficient data to determine if there was any effect on nasal symptoms and to allow power calculations for any further studies.

## Results

### Efficacy of the Iota-Carrageenan nasal spray

35 subjects were screened, enrolled and randomized. One subject (Iota-Carrageenan) was lost in the follow up after the initial screening visit and no additional data were obtained. 34 subjects completed the study drug administration and were included in the safety analysis. Demographic data and a study flow chart are shown in Figure 1. Two subjects were excluded from the analysis of symptoms due to protocol violations that were defined as an exclusion criterion in the study protocol. Subject 11 reported vomiting, nausea and abdominal pain presumably caused by an infection of the gastrointestinal tract and furthermore used ibuprofen as concomitant medication. Subject 23 reported migraine that was treated with ibuprofen and a swollen eye due presumably to an allergic reaction that was treated with an oral anti-histamine during the observation period. The subject was therefore excluded from the efficacy analysis of symptoms. In total, 14 Iota-Carrageenan patients and 18 placebo patients were eligible for analysis of symptoms.

The predefined primary efficacy parameter for the trial was the difference between Iota-Carrageenan and placebo in total symptom scores on days 2-4 (TSS 2-4). Iota-Carrageenan nasal spray was superior to placebo (p < 0.046) with respect to the primary endpoint mean of TSS over days 2-4 (Table 1).

**Table 1:** Study endpoints based on symptoms

End point	Carrageenan nasal spray n = 14	Placebo nasal spray n = 18	p-value
Primary endpoint			
TSS mean of sum on days 2-4	4.62 ± 2,06	6.28 ± 2.29	0.046

Secondary endpoints			
LSS mean of sum on days 2-4	3.79 ± 2,03	5.22 ± 2.30	0.068
SSS mean of sum on days 2-4	0.83 ± 0,75	1.06 ± 1,07	0.704

Shown are symptom scores TSS (total symptom score), LSS (local symptom score), and SSS (systemic symptom score) ± standard deviation.

**Figure 2:**
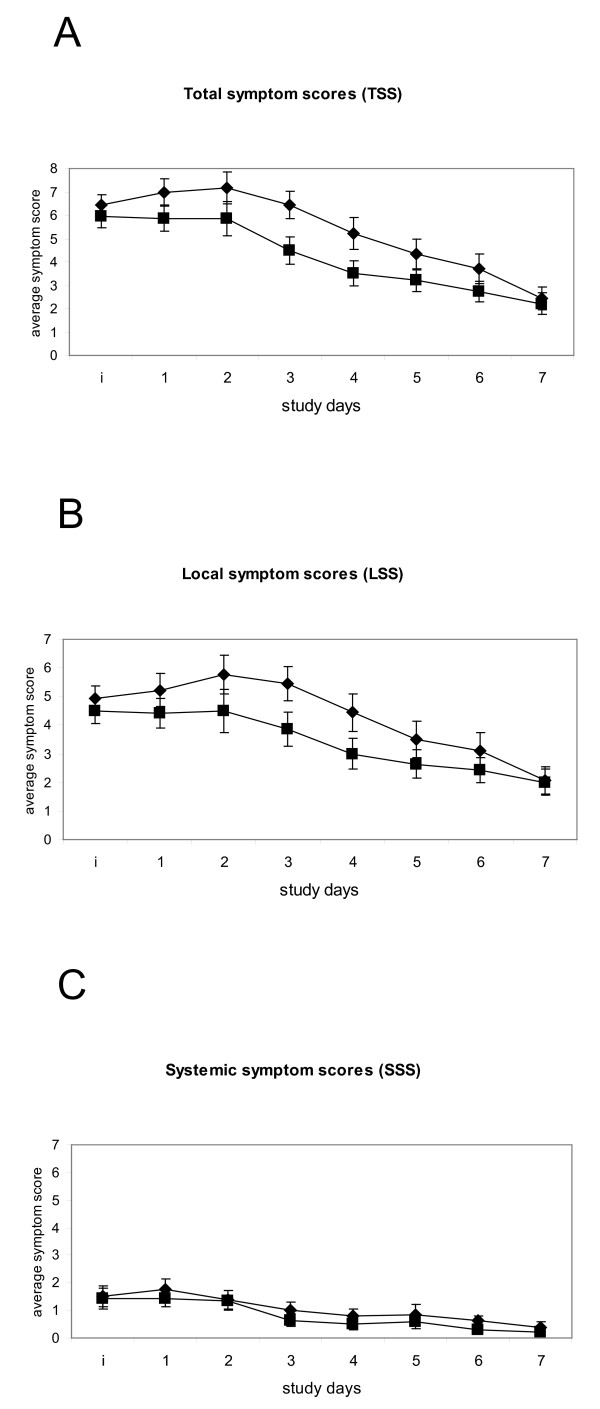
**Mean symptom scores over 7 days**. Mean ± SEM for Carrageenan nasal spray (black squares) and Placebo (black triangles) treatment groups. A. Total symptom scores B. Local Symptom scores C. Systemic symptom scores. The y axis shows the study day; i indicates the point of inclusion into the study.

The mean total symptom scores over 7 days are shown in Figure 2. The efficacy of the Iota-Carrageenan nasal spray treatment appears to be mainly observable on the local symptom scores (LSS) sore throat, blocked nose, runny nose, cough, and sneezing, as there was little difference between treatments for the systemic symptom scores (SSS) headache, muscle ache, chilliness (Figure 2). This is reflected in the results of LSS and SSS mean of sum on days 2-4 which were lower for Iota-Carrageenan patients with significance levels of 0.064 and 0.704, respectively (Table 1). While there is no statistical difference TSS on day 1 (p < 0.231), the difference is significant on day 3 (p < 0.040) indicating a faster relief of symptoms in the Iota-Carrageenan group.

**Figure 3:**
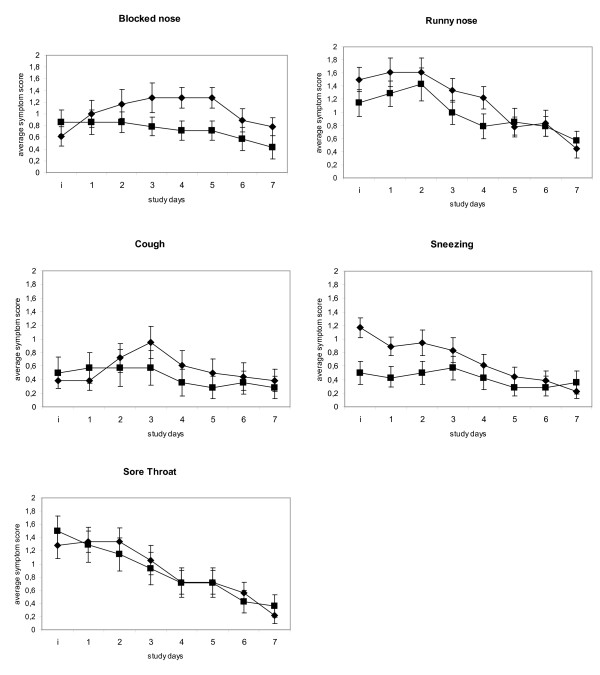
**Mean individual symptom scores over 7 days**. Mean individual symptom scores ± SEM for Carrageenan nasal spray (black squares) and Placebo (black triangles) treatment groups. The y axis shows the day of recording. The y axis shows the study day; i indicates the point of inclusion into the study.

Average individual symptoms scores blocked nose, runny nose, cough, and sneezing were higher for placebo when compared to verum (Figure 3). The individual symptoms blocked nose and runny nose show the highest scores indicating that these symptoms are most bothersome. For all individual LSS a trend towards superiority of the Iota-Carrageenan nasal spray can be observed. At inclusion subjects reported a slightly higher score for the Iota-Carrageenan group for the symptoms blocked nose, cough and sore throat. These three symptoms showed an increase in the placebo group but not in the Iota-Carrageenan group at the next reporting time points. This result suggests that the Iota-Carrageenan nasal spray treatment has an inhibitory effect on the development of common cold symptoms shortly after start of therapy.

At the study end point (day 7), placebo patients reported a mean blocked nose score of 0.78. In contrast, Iota-Carrageenan patients reported a mean blocked nose score of 0.42, corresponding to a reduction of approximately 50% (Figure 3). Further post hoc analysis of this symptom revealed that 71.4% of Iota-Carrageenan patients did not report the symptom blocked nose at the end of the study. In the placebo group only 36.4% of subjects were free of this symptom.

### Antiviral efficacy

Nasal lavages were analyzed by quantitative real time RT-PCR for the presence of viral genomes. Samples of 6 Iota-Carrageenan and 5 placebo patients were virus positive. 5 patients tested positive for human rhinovirus, another 5 patients for coronavirus, and one patient for parainfluenza 3 virus.

**Figure 4:**
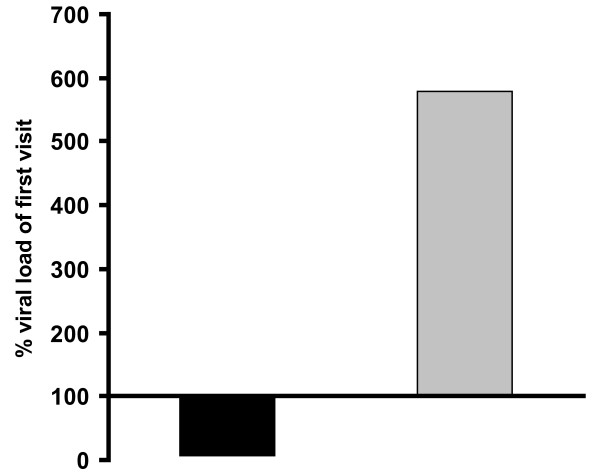
**Relative viral load at day 3/4 in % of day 1**. Shown is the relative viral load on day 3/4 in percent of the viral load on day 1. The mean of the ct values at visit 1 was set 100% for both Iota-Carrageenan and placebo and the percent of the ct values on day 3/4 was calculated as described in materials and methods. The ct numbers of Iota-Carrageenan and placebo samples of day 1 and day 3/4 were compared by applying a Mann-Whitney U-test (p = 0.009). The black bar shows Iota-Carrageenan and the grey bar shows placebo.

As shown in Figure 4, viral load in the placebo group increased almost 6- fold (579%), while it dramatically decreased by 92% in the Iota-Carrageenan group (p < 0.009). This result indicates that the treatment of patients with Iota Carrageenan nasal spray leads to a highly statistically significant reduction of viral load in the nasal cavity, while placebo treatment has no influence on viral replication at all. The basis for the statistical analysis and the ct-values are shown in Additional file 2: Table S2. Nasal lavages of both patients that were excluded due to protocol violations were tested negative for respiratory viruses (data not shown).

### Analysis of cytokines in nasal lavages

The analysis of cytokines revealed that the median level of the following cytokines was below the detection limit of 3,2 pg/ml: EGF, Eotaxin, GM-CSF, IFN-γ, IL-12(p70), IL-13, IL-15, IL-17, IL-1β, IL-2, IL-4, IL-5, IL-6, IL-9, MCP-3, MDC, MIP-1α, MIP-1β, sCD40L, sIL-2Rα, TGFα, TNF-α, TNF-β, and VEGF. The relatively low level of these parameters suggested no major biological relevance at the time point of sampling and consequently no further analysis of above cytokines was carried out.

Cytokine IP-10 (CXCL10) was found to be present in the highest concentration of all tested cytokines. While there was a decrease from 1790 pg/ml at the first visit to an average of 970 pg/ml on day 3/4 in the Iota-Carrageenan group there was an increase to 3016 pg/ml in the placebo group from day 1 to day 3/4 (Table 2). However, due to a high standard deviation the difference of IP-10 levels between Iota-Carrageenan and placebo is not significant. At a much lower level a similar effect was observed for GRO, G-CSF, IL-8, IL-1α, IL-10, IFN-2α and the difference was even significant for FGF-2 (p = 0,04) and Fractalkine (p = 0,023). In contrast, two molecules that are known for their function as antagonists of inflammation, IL-1rα and IL-12(p40), were higher in the Iota-Carrageenan group. However, the result allows hypothesizing that the observed reduction in viral replication resulted in a lower level of pro-inflammatory cytokines and consequently in a lower symptom score.

**Table 2:** Analysis of cytokines in nasal lavages

	first visit day 1	second visit day 3/4	second visit day 3/4	
		
	all	Iota-Carrageenan	Placebo	p-value
FGF-2	5.9 (5.9)	2.5 (2.8)	7.5 (5.6)	0.04

Fractalkine	87.0 (74.5)	46.4 (32.4)	79.7 (39.3)	0.023

GRO	252 (233)	156 (112)	339 (417)	n.s.

G-CSF	45.5 (89.1)	10.9 (17.9)	78.5 (186)	n.s.

IL-8	18.7 (30.6)	14.4 (10.8)	21.0 (22.4)	n.s.

IL-1α	35.3 (30.0)	28.8 (14.4)	43.1 (28.9)	n.s.

IP-10	1790 (3177)	970 (1769)	3016 (4033)	n.s.

IL-10	1.6 (4.22)	0 (0)	5.5 (13.6)	0.049

IL-1ra	164 (129)	174 (320)	131 (85)	n.s.

IFN-α2	11.6 (8.0)	8.7 (4.1)	11.5 (5.6)	n.s.

IL-12(p40)	10.6 (14.0)	9.1 (9.7)	8.0 (9.8)	n.s.

Comparison of cytokine levels on day 1 and day 3/4. Shown are mean levels in pg/ml ± standard deviation. Lower quantification limit was 3.2 pg/ml, values below were set to '0'. P-values: comparison verum versus placebo by Mann-Whitney U-test.

### Product acceptability

Product acceptability by subjects for Iota-Carrageenan and placebo nasal spray, respectively, was significantly higher for Iota-Carrageenan in ITT (p = 0.041), PP (p = 0.009) and also for virus positive patients (p = 0.005) (Table 3). In PP only 1 subject (7%) using Iota-Carrageenan but 6 subjects using placebo (33%) opposed future consistent medication. It is of note that the placebo formulation was identical to verum except Iota-Carrageenan, showing that all components of Iota-Carrageenan nasal spray are exceptionally well tolerated by the subjects, remarkably with even increasing support by virus-positive subjects. These results indicate that the observed benefit of Iota-Carrageenan-treated patients both on the levels of symptom scores and biomarkers correlates with a higher product acceptability.

**Table 3:** Assessment of subject acceptability

	ITT (n = 34)		PP (n = 32)		Subgroup (n = 11)	
	**Verum (n = 16)**	**Placebo (n = 18)**	**Verum (n = 14)**	**Placebo (n = 18)**	**Verum (n = 6)**	**Placebo (n = 5)**

Mean (SD)	6.63 (2.06)	5.39 (2.25)	7.07 (1.69)	5.39 (2.25)	7.50 (0.84)	4.20 (2.49)
p-Value	0.041		0.009		0.005	

Assessment of subject acceptability of Iota-Carrageenan vs. placebo nasal sprays in intention to treat (ITT), per protocol (PP) and subgroup (virus positive) analysis using a VAS scale (0- 10). P-values come from Mann-Whitney U-test. Unit: cm ± standard deviation.

### Safety and tolerability

No serious adverse events (SAE) were reported and there were no withdrawals due to adverse event (AE) development. AEs were listed by patients including the reported term by the investigator, the MedDRA Preferred Term (PT) and the MedDRA System Organ Class (SOC) [16]. 5 subjects (4 Iota-Carrageenan, 1 placebo) experienced at least one AE. Iota-Carrageenan patient 11 reported three AEs - vomiting, nausea and abdominal pain - used ibuprofen as concomitant medication and was therefore excluded from the efficacy analysis of symptoms. The AEs were not considered to be associated with the study medication. Iota-Carrageenan patient 23 reported migraine and puffy eye lids, used ibuprofen and anti-histamine as concomitant medication and was therefore excluded from the efficacy analysis of symptoms. One Iota-Carrageenan patient reported a loss of voice and another Iota-Carrageenan patient reported a dry mouth. Intermittent epistaxis was reported by a placebo patient. A total of 8 AEs were reported, 2 were rated as possibly related to treatment: dry mouth (Iota-Carrageenan, n = 1) in the ITT and PP groups and puffy eye lids (Iota-Carrageenan, n = 1) (Additional file 3: Table S3). Since all side effects were resolved, no special actions were necessary. The small number of AE reports supports in particular the good safety-profile of Iota-Carrageenan as an active agent and Iota-Carrageenan nasal spray components in general.

## Discussion

The results of this study indicate that the Iota Carrageenan nasal spray is a safe and effective treatment when taken within 48 hours of development of common cold symptoms. Designed as an exploratory trial, the size of the study was relatively small but reached statistical significance (p = 0.046) for the predefined primary endpoint (TSS mean of sum on days 2-4). All patients reported relatively low levels of systemic symptoms indicating that no severe infection of the respiratory tract had occurred (Figure 2). The efficacy of the Iota-Carrageenan nasal spray treatment appears to be mainly dependent on the local symptom scores (LSS) sore throat, blocked nose, runny nose, cough, and sneezing (Table 1, Figure 3).

Interestingly, 63.6% of placebo and only 28.6% of Iota-Carrageenan patients reported the symptom blocked nose at the end of the study period. Although not explicitly tested, we conclude that the above fact is one of the main reasons why there was a significantly better acceptability in patients with the Iota-Carrageenan nasal spray (Table 3).

Both the Iota-Carrageenan and the placebo group reached a mean TSS level of around 2 at the end of the 7 day observation period (Figure 2). The study medication was applied only for the first 4 days. This could be a reason why a complete relief of symptoms did not occur in the study period of 7 days. We conclude that both a longer treatment and observation period should be considered in future trials for better determination of the therapeutic effect of the Iota-Carrageenan nasal spray.

The study population consisted mainly of students with a mean age of 19.6 (SD 1.2) years, and the compliance was very high. Since this age group reflects only a small proportion of the general population, this study might serve as a best case indicator for the design of bigger trials targeting the population in general. It is well known from studies with other antiviral substances that early intervention correlates with efficacy. The vast majority of patients (>88%) reported symptoms for 1 day or less on the day of inclusion into the study (Additional file 1: Table S1). We conclude that treatment of common cold with Iota-Carrageenan nasal sprays is effective when started early after onset of symptoms.

Iota-Carrageenan nasal spray is formulated as a solution of Iota-Carrageenan and NaCl in water intended for direct intranasal application. Tests for effectiveness of blinding of the study medication were not carried out. Although the study medication of both groups appeared completely identical this might be a weakness of the study. Independent reviews of randomised controlled clinical trials on upper respiratory tract infection show limited evidence for a benefit of saline nasal irrigation. However, the use of this treatment is widely accepted and some trials obtained satisfactory results [17-19]. The nasal cavity is the site of choice for inhibition of common cold virus infection and replication. The Iota-Carrageenan effects are complemented by the known efficacy, safety and patient satisfaction of saline nasal irrigation in acute or chronic rhino sinusitis via the NaCl/WFI spray component that served as placebo in this study.

The symptomatic benefit for Iota-Carrageenan patients correlated well with the decrease of detectable virus genome copies in nasal lavages of patients (Figure 4). The statistical analysis of the 11 virus positive patients revealed a p-value of 0.009 for the difference between the Iota-Carrageenan nasal spray and the placebo. Since the number of virus positive-tested subjects was low, further confirmation of this result is needed. It cannot be ruled out that some patients were infected with respiratory viruses that were not tested or were below the detection limit. However, this result in patients further supports earlier in-vitro findings of the antiviral effect against human rhinoviruses [10] and against other viruses such a papillomaviruses or dengue virus [11,13]. The results of this study might encourage clinical developments for these viruses as well.

Common cold symptoms are caused by the reaction of the immune system against viruses and virus infected cells as well as local and newly recruited immune cells. In nasal lavage samples the presence of immune mediators was tested. While the majority of the growth factors and cytokines were expressed below the detection limit, 11 mediators including IL-8, IP10, and GRO were easily detectable. It is interesting to note that the expression of the majority of the molecules (FGF-2, Fractalkine, GRO, G-CSF, IL-8, IL-1α, IP-10, IL-10, and IFN-α2) was reduced in the Iota-Carrageenan group upon treatment, while IL-1 receptor antagonist and IL-12p40 were increased. IL-1 receptor antagonist is regarded as counter-acting molecule to IL-1. The role of IL-12p40 during a respiratory infection in humans is not fully understood. A recent study suggested suggest that endogenous IL-12p40 is essential for inhibition of airway hyperresponsiveness and peribronchial fibrosis, but not eosinophilic inflammation, in a murine asthma model with prolonged antigen exposures [20].

The above results suggest that the treatment with Iota-Carrageenan reduces the viral replication. Consequently fewer cells are infected, the immune reaction against the viruses is less pronounced and fewer symptoms occur. In addition, it is reported that the expression of pro-inflammatory mediators in the course of a common cold may worsen pre-existing co-morbidities such as asthma or COPD [6,21,22]. Therefore, a reduction of the immune response due to lower viral load appears as an attractive property of this novel treatment.

The Iota Carrageenan nasal spray used in this study may reduce the severity of nasal symptoms by an antiviral effect rather than any pharmacological effect on nasal blood vessels and glands. This has some advantages as pharmacological interventions to control symptoms such as nasal decongestants and antisecretory agents are associated with side effects such as nasal bleeding and crusting [21].

Young children with respiratory symptoms are major spreaders of rhinovirus in the family setting. Rhinovirus infections are a common cause of hospitalization of children, most often because of wheezing [23,24]. As the vast bulk of viral transmission occurs among children and families an intervention affecting the transmission would be of great socioeconomic value [25]. The lack of toxicity and pharmacological activity of the Iota Carrageenan nasal spray with its high safety profile means that this treatment may be suitable for use in children as well as adults.

## Conclusions

Iota-Carrageenan nasal spray appears to be a promising compound for safe and effective treatment of early symptoms of common cold. Larger clinical trials are needed to study the therapeutic index in more detail.

## Competing interests

The trial was funded by Marinomed Biotechnologie GmbH. REC and MJA did not receive any direct payments from Marinomed. The authors EPG, AGR, CME, and RWE are employed by Marinomed. Authors AGR and EPG are co-founders of Marinomed. AGR and EPG are co-inventors on patent # WO2008067982 held by Marinomed Biotechnologie GmbH that relates to the content of the manuscript. Marinomed Biotechnologie GmbH is financing the processing charge of this manuscript.

## Authors' contributions

REC was principal investigator of the study and was responsible for the study and protocol design. MJA performed the study on site and served as medical director. RWE and CME performed the quantitative virus analysis and the cytokine analysis. AGR, EPG, MJA, REC participated in the design, statistical analyses and coordination of the study, interpretation of data and writing the manuscript. All authors read and approved the final manuscript.

## Supplementary Material

Additional file 1 **Table S1 - Analysis of days of onset of common cold symptoms**. Shown are the numbers of patients for verum, placebo and total divided into groups with days of onset of common cold symptoms at the point of inclusion into the study. P-value comes from Chi square test. Click here for file

Additional file 2 **Table S2 - Viral load of identified viruses, lavage on study day 1 and 3 or 4**. Shown are ct-values of real time PCR: ct values of 35 - 40 indicative for minimal amounts of target viral nucleic acid, cts 30 - 35 for moderate amounts, cts < 30 mark strong positive reactions P-values: comparison verum versus placebo by Mann-Whitney U-test. Unit: ct value. Click here for file

Additional file 3 Table S3 - Summary table of adverse events. Click here for file
