# Supplementary material for: Efficacy and safety of an antiviral Iota-Carrageenan nasal spray: a randomized, double-blind, placebo-controlled exploratory study in volunteers with early symptoms of the common cold
Source: Respir Res. 2010 Aug 10;11(1):108. doi: 10.1186/1465-9921-11-108 (PMC2923116; doi:10.1186/1465-9921-11-108)
Supplement: Additional file 1 — Table S1 - Analysis of days of onset of common cold symptoms. Shown are the numbers of patients for verum, placebo and total divided into groups with days of onset of common cold symptoms at the point of inclusion into the study. P-value comes from Chi square test. [file 1465-9921-11-108-S1.DOC]

### Additional file 1 – Analysis of days of onset of common cold symptoms

Shown are the numbers of patients for verum, placebo and total divided into groups with days of onset of common cold symptoms at the point of inclusion into the study. P-value comes from Chi square test.

|  | Verum | | Placebo | | Total | |
| --- | --- | --- | --- | --- | --- | --- |
| Days | N | % | n | % | n | % |
| 0 | 4 | 25.0 | 5 | 27.8 | 9 | 26.5 |
| 1 | 10 | 62.5 | 11 | 61.1 | 21 | 61.8 |
| 2 | 2 | 12.5 | 2 | 11.1 | 4 | 11.8 |
| Sum | 16 | 47.1 | 18 | 52.9 | 34 | 100 |
| p-value | 0.873 | | | | | |
