# Supplementary material for: Efficacy and safety of an antiviral Iota-Carrageenan nasal spray: a randomized, double-blind, placebo-controlled exploratory study in volunteers with early symptoms of the common cold
Source: Respir Res. 2010 Aug 10;11(1):108. doi: 10.1186/1465-9921-11-108 (PMC2923116; doi:10.1186/1465-9921-11-108)
Supplement: Additional file 2 — Table S2 - Viral load of identified viruses, lavage on study day 1 and 3 or 4. Shown are ct-values of real time PCR: ct values of 35 - 40 indicative for minimal amounts of target viral nucleic acid, cts 30 - 35 for moderate amounts, cts < 30 mark strong positive reactions P-values: comparison verum versus placebo by Mann-Whitney U-test. Unit: ct value. [file 1465-9921-11-108-S2.DOC]

## Additional file 2 – Viral load of identified viruses, lavage on study day 1 and 3 or 4

Shown are ct-values of real time PCR: ct values of 35 – 40 indicative for minimal amounts of target viral nucleic acid, cts 30 – 35 for moderate amounts, cts < 30 mark strong positive reactions P-values: comparison verum versus placebo by Mann-Whitney U-test. Unit: ct value.

|  | Lavage day 1 | | | Lavage day 3 or 4 | | |
| --- | --- | --- | --- | --- | --- | --- |
|  | Verum | Placebo | Total | Verum | Placebo | Total |
| Mean | 30.70 | 31.06 | 30.82 | 34.36 | 28.53 | 31.44 |
| S.D. | 3.99 | 2.74 | 3.33 | 3.10 | 3.97 | 4.55 |
| Median | 29.49 | 31.06 | 29.82 | 33.00 | 30.25 | 32.28 |
| L.Quartile | 27.95 | 29.12 | 28.46 | 32.79 | 25.76 | 30.25 |
| U.Quartile | 33.44 | 33.00 | 33.00 | 33.63 | 31.58 | 33.00 |
| Minimum | 27.44 | 29.12 | 27.44 | 32.50 | 22.99 | 22.99 |
| Maximum | 36.37 | 33.00 | 36.37 | 39.86 | 32.05 | 39.86 |
| n = | 4 | 2 | 6 | 5 | 5 | 10 |
| p-value | 0.643 | | | 0.009 | | |
