# Supplementary material for: Efficacy and safety of an antiviral Iota-Carrageenan nasal spray: a randomized, double-blind, placebo-controlled exploratory study in volunteers with early symptoms of the common cold
Source: Respir Res. 2010 Aug 10;11(1):108. doi: 10.1186/1465-9921-11-108 (PMC2923116; doi:10.1186/1465-9921-11-108)
Supplement: Additional file 3 — Table S3 - Summary table of adverse events. [file 1465-9921-11-108-S3.DOC]

## Additional file 3 – Summary table of adverse events

| No. | Type | AE | Comedication |
| --- | --- | --- | --- |
| 6 | Verum | loss of voice | none |
| 11 | Verum | vomiting |  |
| 11 | Verum | nausea | ibuprofen |
| 11 | Verum | abdominal pain |  |
| 18 | Verum | dry mouth | none |
| 21 | Placebo | epistaxis | none |
| 23 | Verum | migraine | ibuprofen |
| 23 | Verum | puffy eye lids | anti-histamine |
